# Effects of remote breastfeeding guidance on breastfeeding rates and neonatal health: a systematic review and meta-analysis

**DOI:** 10.3389/fpubh.2026.1696927

**Published:** 2026-05-01

**Authors:** Miaomiao Hu, Sang Sang, Hui Pei, Xiao Han, Qiqi Wei, Li Wei, Ji Qi, Jing Li

**Affiliations:** 1 Department of Neonatology, The Second Affiliated Hospital of Shandong First Medical University, Taian, China; 2 Department of Pediatric Surgery, The Second Affiliated Hospital of Shandong First Medical University, Taian, China; 3 Department of Cardiology, The Second Affiliated Hospital of Shandong First Medical University, Taian, China; 4 Department of Pediatrics, Heze Municipal Hospital, Heze, China; 5 Department of Pediatrics, The Second Affiliated Hospital of Shandong First Medical University, Taian, China

**Keywords:** breastfeeding, meta-analysis, randomized controlled trials, remote breastfeeding guidance, trial sequential analysis

## Abstract

**Background:**

Breastfeeding is widely acknowledged as the optimal feeding method for neonatal health. Remote breastfeeding guidance (e.g., using the telephone, text messages, mobile applications, and so on) may improve breastfeeding rates and neonatal outcomes, but its short-term impact and effect across economic regions remain unclear. This systematic review and meta-analysis aimed to assess the effect of remote breastfeeding guidance on neonatal feeding practices and breastfeeding rates.

**Methods:**

Databases including PubMed, Embase, and the Cochrane Library were screened from inception to November 2024 to identify randomized controlled trials (RCTs) that assessed the effects of remote breastfeeding guidance on breastfeeding rates and neonatal health. Time subgroup analyses evaluated exclusive and any breastfeeding at 3 and 6 months postpartum, together with the effects in developed and less developed regions. Trial Sequential Analysis (TSA) and sensitivity analysis were employed to address heterogeneity and evaluate the robustness of the findings.

**Results:**

Thirty RCTs involving 8,389 infants were incorporated into this systematic review and meta-analysis. Compared to the control group, remote breastfeeding support significantly increased the prevalence of exclusive breastfeeding at 3 and 6 months (*RR* = 1.17, 95% CI 1.11–1.23, *P* < 0.0001; *RR* = 1.57, 95% CI 1.38–1.77, *P* < 0.0001), with modest effects on any breastfeeding (*RR* = 1.07, 95% CI 1.02–1.13, *P* = 0.007; *RR* = 1.05, 95% CI 0.99–1.11, *P* = 0.11). Infant weight was significantly higher in the intervention groups at 3 and 6 months (*MD* = 334.39, 95% CI 310.93–357.85, *P* < 0.00001), representing clinically meaningful gains. A subgroup analysis demonstrated that exclusive breastfeeding provided greater benefits in less developed regions (*RR* = 1.28, 95% CI 1.23–1.34, *P* < 0.00001) than in developed regions (*RR* = 1.12, 95% CI 1.05–1.19, *P* < 0.00001). TSA and sensitivity analyses confirmed the robustness of the results.

**Conclusions:**

Remote breastfeeding guidance plays an important role in increasing breastfeeding rates and promoting physical development, with particularly pronounced effects in less developed regions.

## Introduction

Breastfeeding is widely endorsed by the World Health Organization (WHO) and neonatal guidelines as the optimal infant feeding practice. Breastfeeding provides essential nutrients, immune protection, and long-term health benefits (1, 2). Despite these established benefits, global breastfeeding rates remain suboptimal and are characterized by significant regional disparities. Recent data have highlighted this challenge: the WHO reported a 6-month exclusive breastfeeding (EBF) rate of 48% worldwide in 2024, approaching but falling short of the 50% target (3). Regional data have further underscored this gap. In Europe, even Baby-Friendly Hospitals (BFHs) in Greece achieved only a 21.2% EBF rate at 6 months (compared to a national average of 0.8%) (4). In mainland China, exclusive breastfeeding rates remain critically low, with only 12.3% of mothers in Jiangsu Province sustaining EBF for 6 months (5). In Africa, South Africa's EBF rate dropped to 22% at 6 months in 2025 (from 32% in 2016) (6). These findings highlight the widespread and urgent need for innovative solutions to bridge the gap between global recommendations and actual practice.

Traditional barriers—such as insufficient maternal education, restricted access to lactation services, maternal employment, and sociocultural constraints—frequently undermine breastfeeding initiation and continuation (7, 8). In recent years, the advent of telemedicine and digital health interventions has led to the development of remote breastfeeding guidance systems to support mothers in breastfeeding and neonatal care (9, 10).Remote breastfeeding guidance involves the use of digital communication technologies, such as phone consultations, video calls, mobile applications, and online support groups, to provide education and assistance in neonatal feeding. Studies have suggested that remote lactation support can increase breastfeeding initiation rates and reduce early weaning. In Nigeria, an RCT (11) involving 194 low-income teenage mothers showed that the BabyThrive application effectively increased maternal awareness of infant and young child feeding practices. However, the effectiveness of such interventions varies depending on geographic, socioeconomic, and healthcare accessibility factors.

This variability underscores the necessity of a comprehensive, updated assessment of whether remote breastfeeding guidance consistently improves neonatal feeding outcomes in diverse settings. Although previous meta-analyses have explored telehealth interventions, significant evidence gaps remain. Most notably, a comprehensive review by Gavine et al. (12) reported modest short-term gains in preventing cessation of EBF at 3 months (*RR* 0.75; low certainty), but found that these effects diminished by 6 months amid substantial clinical heterogeneity. Furthermore, prior reviews largely neglected critical neonatal physical development outcomes and lacked targeted subgroup comparisons based on regional economic development. Consequently, the sustained efficacy of remote support and its optimal application in resource-constrained environments remain inadequately quantified.

To address these critical limitations, we conducted an updated systematic review and meta-analysis to evaluate the impact of remote breastfeeding guidance on breastfeeding rates at 3 and 6 months postpartum. As secondary outcomes, we also assessed neonatal health parameters, including infant weight and growth metrics. Given the vast global disparities in healthcare infrastructure, we hypothesized that the efficacy of remote support would differ by region; therefore, we pre-specified subgroup analyses that stratified studies into those in developed and those in less developed countries. Finally, to ensure the robustness and reliability of our pooled results, we incorporated Trial Sequential Analysis (TSA) to determine if the current cumulative evidence is statistically conclusive (13). By rigorously aggregating recent RCT data, this study aims to provide definitive, region-specific evidence to inform scalable digital health policies for maternal and child healthcare systems worldwide.

## Methods

### Study design

This systematic review and meta-analysis was prospectively registered with the International Prospective Register of Systematic Reviews (PROSPERO: CRD420251028166). It was conducted following the PRISMA guidelines and the Cochrane Handbook for Systematic Reviews of Interventions. The study aimed to assess the effects of remote breastfeeding guidance on neonatal feeding practices and breastfeeding rates, including exclusive breastfeeding or any breastfeeding at 3 or 6 months postpartum. The registered protocol was followed without major deviations.

### Search strategy

A comprehensive literature search was conducted across multiple databases, including PubMed, Embase, and the Cochrane Library, from database inception to November 2024. The search strategy combined Medical Subject Headings (MeSH) or Emtree terms with free-text words. The search concepts included three main domains: (1) breastfeeding (e.g., “Breast Feeding”, “Breast Milk”); (2) target population (e.g., “Infant”, “Newborn”); and (3) remote guidance interventions, which were broadly expanded to include synonyms such as “telehealth”, “telemedicine”, “remote consultation”, “mHealth”, “eHealth”, “telephone”, “smartphone”, “mobile application”, “text messaging”, and “internet”. No language restrictions were applied during the search process. The detailed search strategies for each database are provided in the Supplementary materials. Additional manual searches were performed by screening the reference lists of identified articles to capture any potentially relevant studies missed during the database searches.

### Eligibility criteria

Remote breastfeeding guidance was broadly defined as any intervention delivered via distance communication technologies (e.g., telephone, SMS/text messages, mobile applications, video conferencing, internet platforms, or combinations thereof) aimed at promoting breastfeeding initiation, duration, or exclusivity. This inclusive definition reflects the heterogeneous nature of the interventions identified in the literature. As a result, our pooled analyses treated remote guidance as a broad category rather than comparing distinct delivery formats.

### Selection of studies and data collection

Two independent reviewers conducted the study selection and data extraction, performing cross-verification at each step to maintain accuracy, reduce bias, and ensure consistency. Full-text articles that potentially met the eligibility criteria were obtained and evaluated. Any disagreements between the reviewers were resolved through discussion or adjudication by a third reviewer. The collected data encompassed study characteristics, participant demographics, details of interventions, and outcome measures.

### Statistical analysis

The meta-analysis was performed utilizing RevMan and Stata software. Dichotomous data were analyzed by calculating relative risks (*RR*), whereas continuous data were analyzed by calculating mean differences (*MD*) with 95% confidence intervals. Study heterogeneity was evaluated using the chi-square (χ^2^) test and *I*^2^ statistics. A fixed-effects model was selected when *I*^2^ < 50% and *P* > 0.10, reflecting low heterogeneity; otherwise, a random-effects model was employed when *I*^2^ > 50% or *P* < 0.10, indicating significant heterogeneity. Sources of heterogeneity were explored through sensitivity analyses. Potential publication bias was evaluated visually through funnel plots and statistically quantified using Egger's linear regression test. A *P* < 0.05 was considered to indicate significant publication bias. For subgroup analyses based on economic development, countries were stratified into “developed” and “less developed” categories using the International Monetary Fund (IMF) classification framework. No adjustments for multiple comparisons were applied to the subgroup analyses, which were pre-specified based on prior literature and clinical rationale.

### Trial sequential analysis (TSA)

Trial Sequential Analysis was applied to limit random errors and confirm the stability of the results. The required information size was calculated based on an anticipated intervention effect, α = 5%, and power = 80%. The cumulative Z-curve was plotted against conventional boundaries and trial sequential monitoring boundaries to determine if sufficient evidence had been reached or if further trials were necessary.

### Risk of bias assessment

The Cochrane Risk of Bias 2.0 tool was used to assess the methodological quality of the RCTs by examining domains such as the randomization process, allocation concealment, blinding, incomplete outcome data, and selective reporting. Each study was classified as having low, some concerns, or high risk of bias. Sensitivity analyses were performed by removing trials with a high risk of bias to explore their effect on the meta-analytic outcomes.

## Results

### Study characteristics

**Figure 1:**
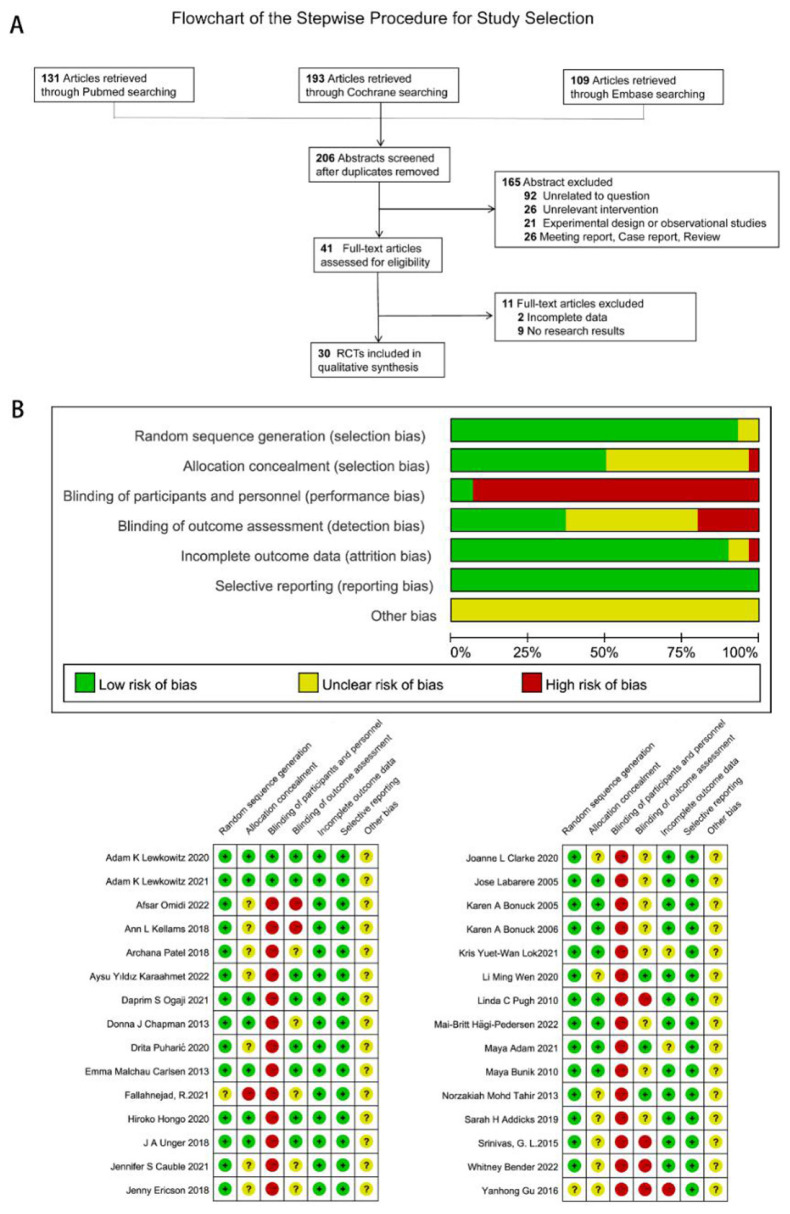
PRISMA flow diagram and risk of bias assessment for the included studies. **(A)**. Flow diagram based on PRISMA guidelines for this systematic review and meta-analysis **(B)**. Risk of bias assessment for all included randomized controlled trials. RCT, randomized controlled trial.

A total of 433 relevant studies were identified in the PubMed/MEDLINE, EMBASE, and Cochrane databases from their inception to November 2024. After removing duplicate literature, 206 studies were considered for the initial screening. After screening the titles and abstracts, 165 studies were excluded during the secondary screening. After a full-text review of the remaining 41 articles, 30 RCTs were selected for analysis in the systematic review and meta-analysis. The PRISMA flowchart detailing the literature retrieval process and the risk assessment results of the RCTs are presented in Figures 1A, B. More detailed descriptions of the included studies' characteristics are available in Supplementary Table 1.

### Neonatal breastfeeding at 3 months

There were 27 RCTs (14–40) included in the meta-analysis on remote breastfeeding guidance for neonatal feeding at 3 months. Sensitivity analysis revealed significant heterogeneity in the studies by Archana Patel (16) and Maya Adam (34), which were excluded from subsequent analyses, as detailed in Supplementary Figures S1A, B. Compared to the control group, remote breastfeeding guidance significantly improved exclusive breastfeeding at 3 months (*RR* = 1.17, 95%CI 1.11–1.23, *P* < 0.00001), while its effect on any breastfeeding was relatively small (*RR* = 1.07, 95%CI 1.02–1.13, *P* = 0.007), as shown in Supplementary Figures S2A, B.

**Figure 2:**
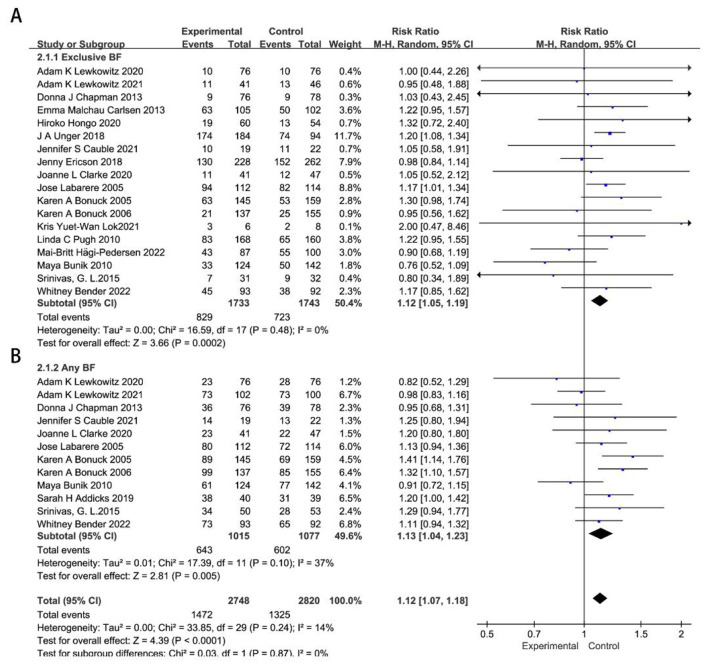
Effects of remote breastfeeding guidance on neonatal feeding practices in developed regions at 3 months. Exclusive breastfeeding **(A)** and any breastfeeding **(B)** rates among participants receiving remote support interventions. RR, risk ratio; CI, confidence interval; Random, random effects model; Fixed, fixed effects model.

**Figure 3:**
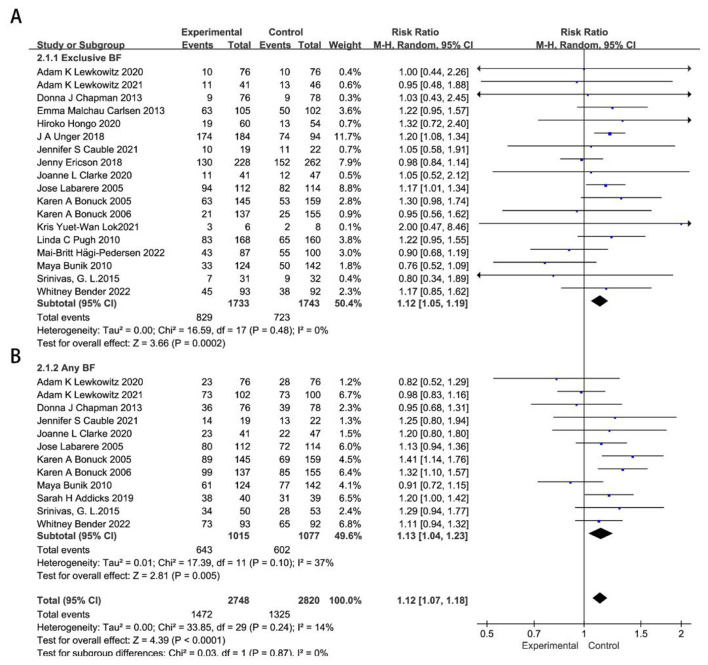
Effects of remote breastfeeding guidance on neonatal feeding practices in less developed regions at 3 months. Exclusive breastfeeding **(A)** and any breastfeeding **(B)** rates among participants receiving remote support interventions. RR, risk ratio; CI, confidence interval; Random, random effects model; Fixed, fixed effects model.

A subgroup analysis based on the economic development of the study regions revealed that remote breastfeeding guidance has a more significant impact on exclusive breastfeeding in less developed regions (*RR* = 1.28, 95%CI 1.23–1.34, *P* < 0.0001), while also showing a significant impact in developed regions (*RR* = 1.12, 95%CI 1.05–1.19, *P* = 0.0002). Remote breastfeeding guidance has a more significant impact on any breastfeeding rates in developed regions than in less developed regions (*RR* = 1.13, 95%CI 1.04–1.23, *P* = 0.005; *RR* = 0.95, 95%CI 0.89–1.01, *P* = 0.09), as shown in Figures 2, 3.

Additionally, the funnel plot and Egger's test presented in the literature after excluding highly heterogeneous studies did not reveal significant publication bias, as detailed in Supplementary Figure S3A. TSA analysis demonstrated that the cumulative Z-curves for the subgroup analysis exceeded both the conventional test boundary and the TSA boundaries, confirming that the study's findings have stabilized, as detailed in Supplementary Figures S4A, B.

### Neonatal breastfeeding at 6 months

A total of 23 RCTs were included in the meta-analysis on 6-month remote breastfeeding guidance for breastfeeding (14–21, 24, 25, 27–32, 34–37, 39, 41, 42). The studies by Archana Patel (16) and Maya Adam (34) were excluded from the subsequent analysis due to significant heterogeneity in the sensitivity analysis, as detailed in Supplementary Figures S1C, D. Remote breastfeeding guidance significantly increased exclusive breastfeeding at 6 months compared to the control group (*RR* = 1.57, 95% CI 1.38–1.77, *P* < 0.0001), whereas it had no statistically significant impact on any breastfeeding (*RR* = 1.05, 95% CI 0.99–1.11, *P* = 0.11), as shown in Supplementary Figures S5A, B.

**Figure 4:**
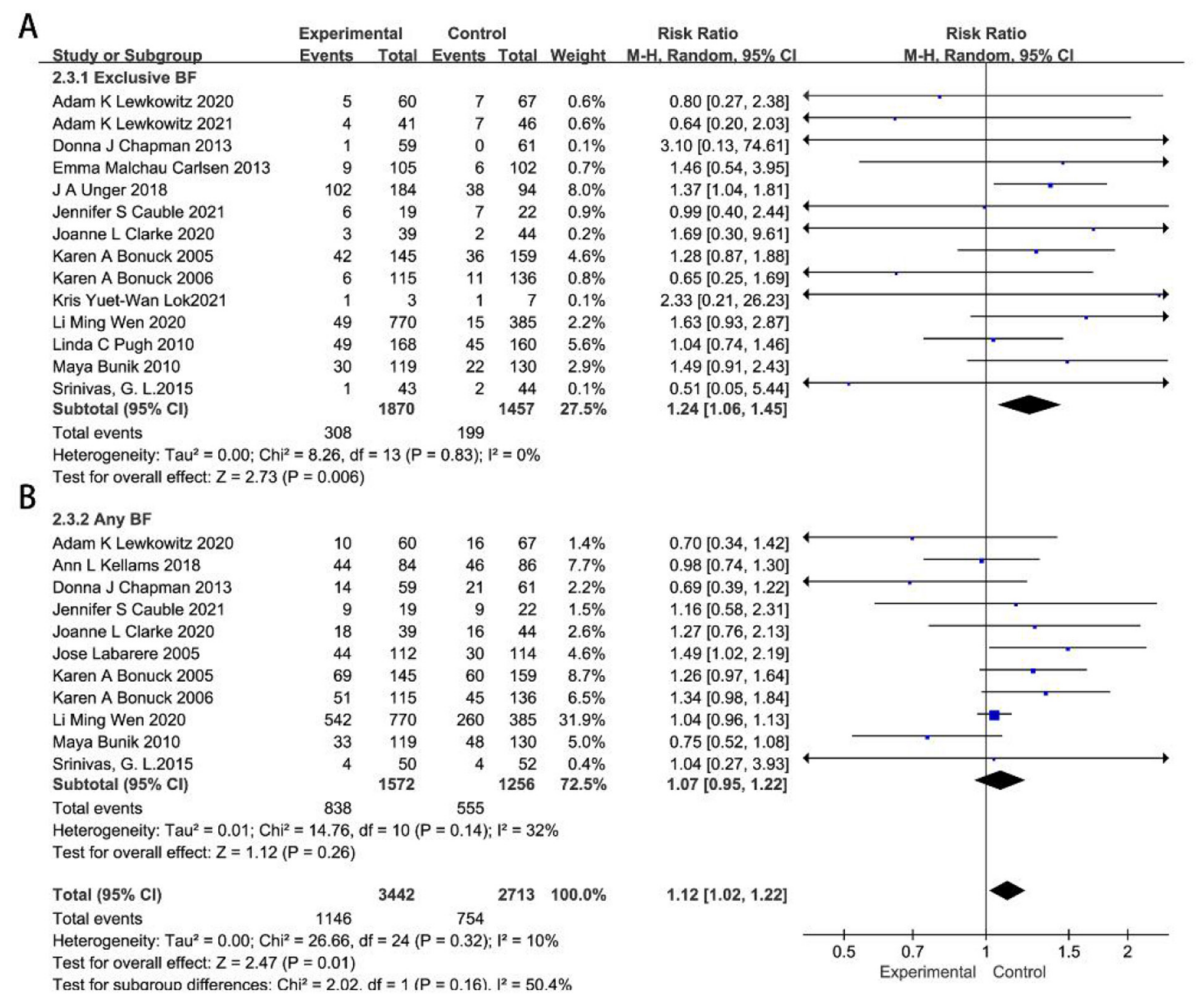
Effects of remote breastfeeding guidance on neonatal feeding practices in developed regions at 6 months. Exclusive breastfeeding **(A)** and any breastfeeding **(B)** rates among participants receiving remote support interventions. RR, risk ratio; CI, confidence interval; Random, random effects model; Fixed, fixed effects model.

**Figure 5:**
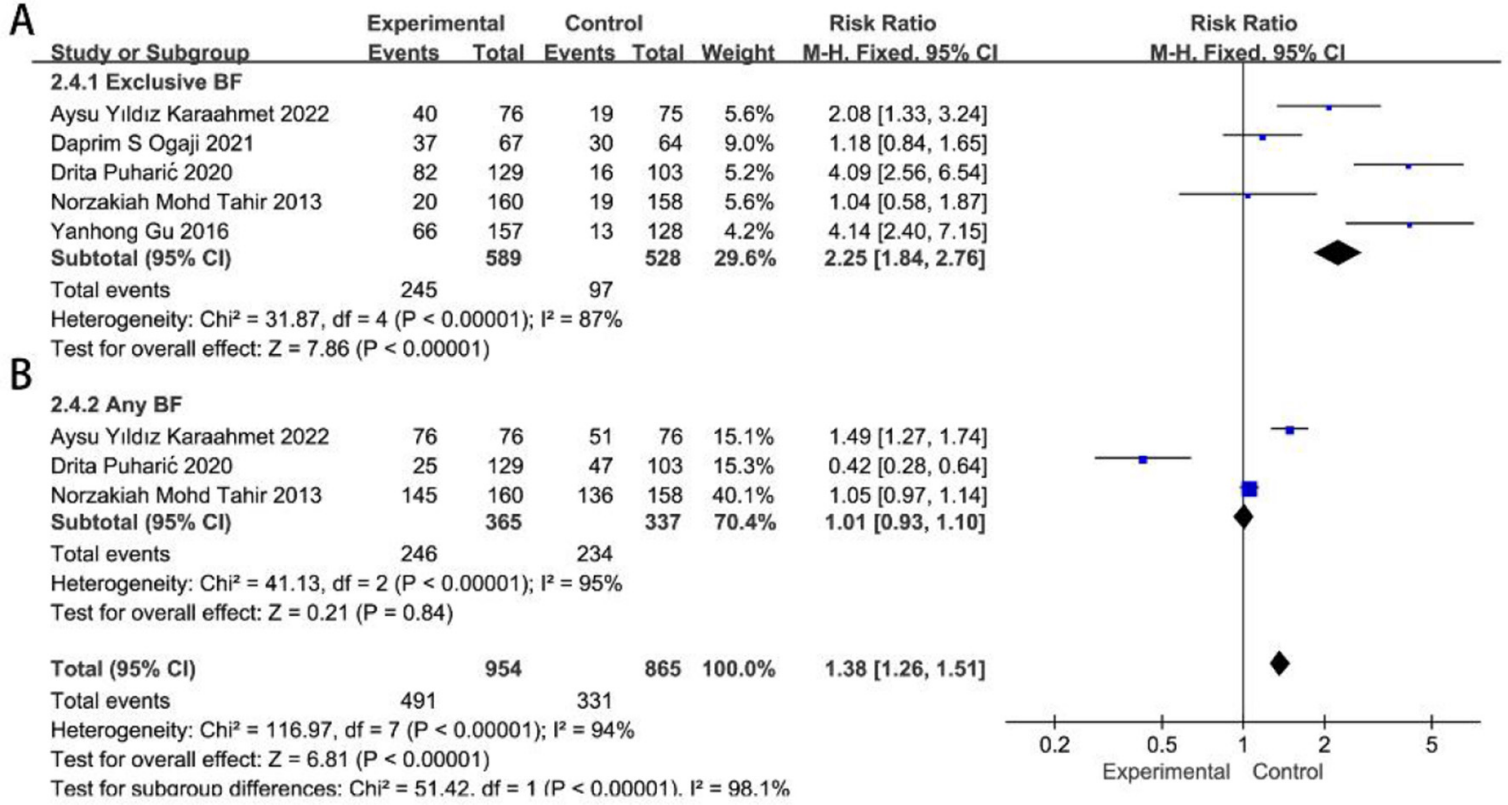
Effects of remote breastfeeding guidance on neonatal feeding practices in less developed regions at 6 months. Exclusive breastfeeding **(A)** and any breastfeeding **(B)** rates among participants receiving remote support interventions. RR, risk ratio; CI, confidence interval; Random, random effects model; Fixed, fixed effects model.

Subgroup analysis demonstrated that remote guidance has a more significant impact on exclusive breastfeeding in less developed regions (*RR* = 2.25, 95%CI 1.84–2.76, *P* < 0.0001), while it had a relatively smaller effect in developed regions (*RR* = 1.24, 95% CI 1.06–1.45, *P* = 0.006). In both developed and less developed regions, remote guidance for neonatal feeding had no statistically significant impact on any breastfeeding (*RR* = 1.07, 95% CI 0.95–1.22, *P* = 0.26; *RR* = 1.01, 95% CI 0.93–1.10, *P* = 0.84), with the results presented in Figures 4, 5.

Analysis of the funnel plot and Egger's test in the literature confirmed the absence of significant publication bias, as detailed in Supplementary Figure 3B. TSA analysis indicates that the meta-analysis of 6-month remote guidance for breastfeeding reached stable outcomes, as detailed in Supplementary Figures S4C, D.

### Physical development

**Figure 6:**
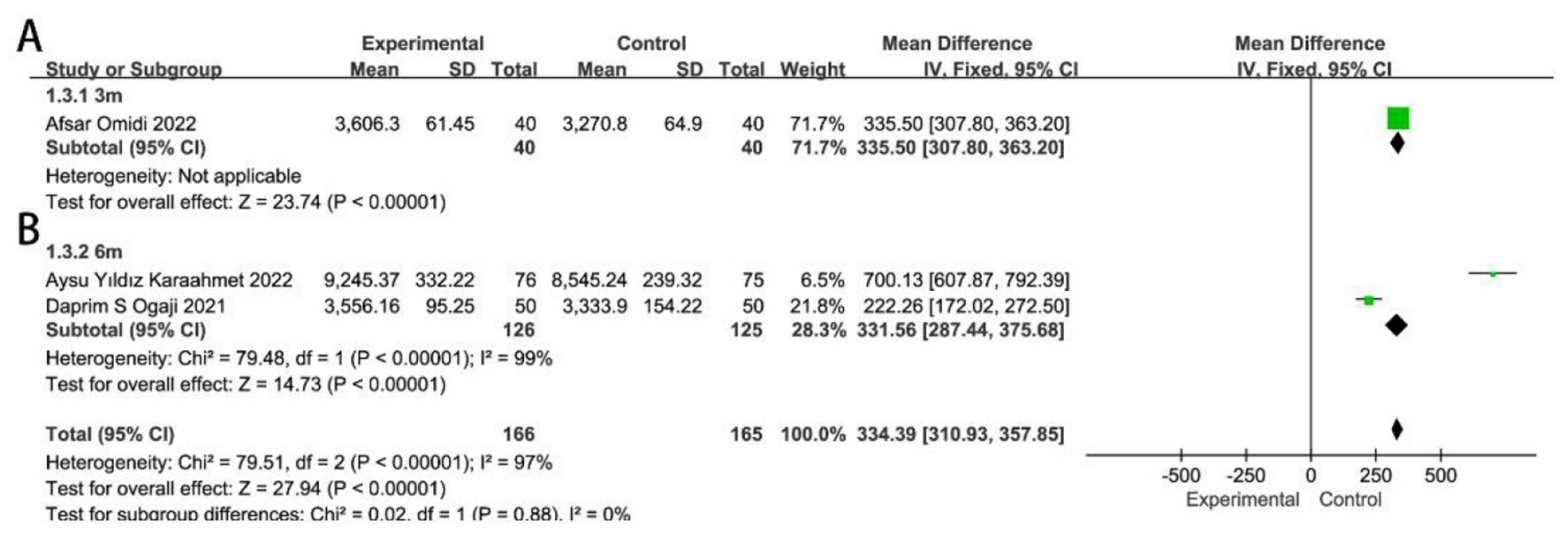
Effects of remote breastfeeding guidance on neonatal weight at 3 months **(A)** and 6 months **(B)**. RR, risk ratio; CI, confidence interval; Random, random effects model; Fixed, fixed effects model.

Only three RCTs (9, 17, 18) were included in the meta-analysis on remote breastfeeding guidance for infant weight. Infant weight increased significantly in remote guidance groups at 3 and 6 months (*MD* = 334.39, 95%CI 310.93–357.85, *P* < 0.00001), as shown in Figure 6. However, with only three RCTs reporting on infant growth parameters, the available data were too limited to perform additional subgroup analyses; therefore, these secondary outcomes warrant cautious interpretation. Karaahmet AY's study (17) found that infants in the remote breastfeeding guidance group had significantly greater height at 6 months (69.89 ± 2.32 cm vs. 65.89 ± 2.32 cm, *P* < 0.001). Similarly, Ogaji DS's study (18) showed that infants receiving remote breastfeeding support have significantly higher growth development scores at 6 months, as measured by Weight-for-Age z-scores (0.40 ± 1.09 vs. −0.03 ± 1.06, *P* = 0.022). Although the studies (20, 35) by Bunik and Puharić D did not observe significant differences in weight or length in the remote guidance group, they did show a significant decrease in the incidence of illness during the first and third months (25% vs. 35%, *P* = 0.05; 7% vs. 24%, *P* = 0.005, respectively).

## Discussion

Breastfeeding has significant physiological, psychological, and social benefits for both mothers and infants, establishing it as a cornerstone of early childhood and maternal health (1, 43, 44). According to WHO guidelines, infants should be exclusively breastfed for the first 6 months, and breastfeeding with complementary foods should continue up to 2 years of age or beyond. Unfortunately, the current global average exclusive breastfeeding rate is only approximately 40%, with exclusive breastfeeding rates in less developed regions significantly below the 70% target (45). Even in some developed countries, such as the United States and Australia, breastfeeding rates fall below the global average (7, 46). The widespread insufficiency in breastfeeding rates and duration is a pressing global concern that demands urgent attention.

Prenatal and postnatal breastfeeding education, feeding training in hospitals and clinics, and remote breastfeeding support are some of the key strategies for effectively increasing breastfeeding rates and improving maternal and infant health (47). In its “Guideline: counselling of women to improve breastfeeding practices,” the WHO encourages the use of various channels and technologies to provide ongoing breastfeeding guidance to mothers. Remote breastfeeding support (including text message reminders, telephone follow-ups, and mobile applications) has great potential to improve breastfeeding sustainability (48). Compared to traditional face-to-face guidance and child healthcare visits, remote breastfeeding support offers significant advantages, including flexibility, sustainability, immediacy, efficiency, and low cost. This approach is especially effective in less developed regions with limited healthcare resources. (49)

The mechanisms underlying the success of remote guidance are likely multifactorial. From a behavioral perspective, frequent remote contact (via proactive calls or messages) acts as a powerful reinforcing cue that improves maternal adherence to WHO feeding guidelines. Psychologically, remote support provides just-in-time reassurance during vulnerable periods, such as when a mother perceives that she does not have enough milk or when her infant has a growth spurt, which directly bolsters maternal breastfeeding self-efficacy and confidence. Unlike episodic in-person clinic visits, the continuous nature of remote guidance helps mothers independently navigate daily challenges, fostering a proactive coping mechanism that is crucial for sustaining exclusive breastfeeding.

This systematic review and meta-analysis confirms that remote support, delivered via telephone, text messages, mobile applications, or video conferencing, can serve as an effective alternative to or complement for in-person care, significantly increasing exclusive breastfeeding rates by approximately 20% to 50% between 3 and 6 months postpartum. A previous comprehensive meta-analysis by Gavine demonstrated that while remote interventions improve exclusive breastfeeding rates at 3 months (*RR* = 0.75 for EBF cessation), this effect dissipates by 6 months amid substantial heterogeneity. In contrast, our updated analysis, which incorporates more recent, post-COVID-19 trials, demonstrates a sustained and even amplified effect at the 6-month mark. This discrepancy may be driven by the evolving sophistication of digital health interventions and their increasing integration into routine postnatal care.

Interestingly, the effect of remote breastfeeding guidance was greater at 6 months than at 3 months. This pattern may be explained by several factors. First, 6 months represents a critical transition period for breastfeeding, as many mothers return to work, introduce complementary foods, and experience reduced breastfeeding self-efficacy at this time point. Remote guidance may be particularly effective at this stage by providing timely emotional support, problem-solving strategies, and practical lactation advice. Second, the baseline rate of breastfeeding is typically lower at 6 months than at 3 months, which increases the potential for relative improvement and thus amplifies the effect size. Finally, some of the included studies may have specifically targeted interventions around the 6-month mark, which could have further enhanced the observed effect at this time point.

The impact of remote support differed between exclusive and any breastfeeding (*RR* = 1.17 vs. 1.07 at 3 months; *RR* 1.57 vs. 1.05 at 6 months). This finding suggests that remote breastfeeding guidance may be more effective for exclusive breastfeeding, as it primarily facilitates early lactation maintenance, breastfeeding technique, and maternal self-efficacy. By contrast, once formula supplementation starts, the continuation of any breastfeeding is influenced by broader social and behavioral factors that are less modifiable through remote interventions, underscoring the importance of providing early preventive support.

Furthermore, this meta-analysis demonstrates that remote breastfeeding guidance has a more pronounced effect in less developed regions, where access to medical infrastructure and lactation consultation services is limited (*RR* = 1.28 vs. 1.12 at 3 m; *RR* = 2.25 vs. 1.24 at 6 m). In countries such as India, Nigeria, and parts of China (16, 18, 39), remote interventions were particularly effective in improving exclusive breastfeeding rates, likely due to the combined influence of lower baseline breastfeeding rates, limited healthcare resource distribution, and inadequate maternal health education. In these contexts, remote support plays a vital role by helping to overcome structural barriers such as geographic distance, transportation difficulties, and shortages of trained healthcare personnel, while also enhancing resource allocation and the quality of postpartum education. By contrast, in more developed settings where in-person lactation support is already widely accessible, remote interventions may only offer incremental benefits, serving more as a supplementary approach rather than a primary component of postpartum care. These findings highlight the potential of low-cost, scalable remote interventions to reduce global disparities in maternal and child health outcomes.

It should be noted that this review did not seek to identify the “best” mode of remote breastfeeding guidance. Many of the included studies did not clearly specify or differentiate between types of remote support, and several interventions combined multiple modalities (mixed digital and phone-based support). As a result, our pooled analyses treated remote guidance as a broad category rather than comparing distinct delivery formats. Furthermore, the limited number of available trials and their heterogeneity limit our ability to conduct meaningful subgroup analyses by mode of delivery. The available evidence for neonatal health outcomes in this review is largely restricted to infant weight and growth-related indicators, with only a limited number of RCTs contributing data. Therefore, the findings should be interpreted cautiously and should not be extended to broader neonatal outcomes such as morbidity, neurodevelopment, or cognitive development.

Sensitivity analysis identified the studies by Archana Patel (16) and Maya Adam (34) as major sources of clinical and methodological heterogeneity due to their critical deviations from standard remote interventions. Specifically, the trial by Maya Adam utilized a mixed-modality approach involving tablet-based videos during in-person home visits, rather than exclusively remote delivery. Conversely, the study by Archana Patel introduced significant socioeconomic bias by directly providing participants with cell phones and free recharge vouchers. Importantly, although including these two studies substantially inflated statistical heterogeneity, it did not alter the positive overall impact on breastfeeding rates.

Future studies with clearly defined, homogeneous intervention types and larger sample sizes could facilitate more targeted comparisons to determine the most effective forms of remote lactation support. Moreover, additional research is needed to evaluate the long-term outcomes of remote breastfeeding guidance, including its potential effects on health, cognitive development, and sustained breastfeeding beyond 6 months. These findings could help inform the development of evidence-based strategies for integrating remote breastfeeding support into routine maternal and child healthcare systems, particularly in less developed countries.

## Limitations

Several limitations of this review should be acknowledged. First, although statistical evaluations indicated no significant publication bias, the potential for such bias cannot be entirely discounted, particularly regarding unpublished negative trials or gray literature. Second, there was considerable variability in intervention intensity (e.g., frequency and duration of follow-ups) and delivery modes across the included studies. Because some interventions combined multiple modalities, our analysis treated remote breastfeeding guidance as a broad category rather than comparing distinct delivery formats, which may affect the generalizability of the results. Finally, the study lacks an assessment of long-term outcomes, so the potential effects on health, cognitive development, and sustained breastfeeding beyond 6 months remain unclear.

## Conclusions

Remote breastfeeding guidance plays an important role in increasing breastfeeding rates and promoting physical development, with particularly pronounced effects in less developed regions.

## Data Availability

The original contributions presented in the study are included in the article/Supplementary material, further inquiries can be directed to the corresponding authors.

## Supplementary material

Supplementary Table S1 Characteristics of remote breastfeeding interventions.

Supplementary Figure S1 Sensitivity analysis of the meta-analysis. **(A)** Exclusive breastfeeding at 3 months; **(B)** any breastfeeding at 3 months. **(C)** Exclusive breastfeeding at 6 months; **(D)**. Any breastfeeding at 6 months.

Supplementary Figure S2 Effects of remote breastfeeding guidance on neonatal feeding practices at 3 months. **(A)** Exclusive breastfeeding; **(B)** Any breastfeeding. RR, risk ratio; CI, confidence interval; Random, random effects model; Fixed, fixed effects model.

Supplementary Figure S3 Funnel plot of remote breastfeeding guidance at 3 months **(A)** and 6 months **(B)**.

Supplementary Figure S4 TSA analysis of the meta-analysis. **(A)** Exclusive breastfeeding at 3 months; **(B)** any breastfeeding at 3 months. **(C)** Exclusive breastfeeding at 6 months; **(D)** Any breastfeeding at 6 months.

Supplementary Figure S5 Effects of remote breastfeeding guidance on neonatal feeding practices at 6 months. **(A)** Exclusive breastfeeding; **(B)** Any breastfeeding. RR, risk ratio; CI, confidence interval; Random, random effects model; Fixed, fixed effects model.
