# Supplementary material for: Effects of remote breastfeeding guidance on breastfeeding rates and neonatal health: a systematic review and meta-analysis
Source: Front Public Health. 2026 May 1;14:1696927. doi: 10.3389/fpubh.2026.1696927 (PMC13176203; doi:10.3389/fpubh.2026.1696927)
Supplement: Supplementary Table S1 — Characteristics of remote breastfeeding interventions. [file Table_1.docx]

Supplementary Table

**Characteristics of Remote Breastfeeding Interventions**

| **Study** | **Method** | **Frequency** | **Duration** | **Main Intervention Content** |
| --- | --- | --- | --- | --- |
| Afsar Omidi  (2022) | Two in-hospital face-to-face educational sessions, followed by three 20-minute remote educational sessions via phone and video conference | Two in-hospital sessions, three remote sessions at health centers | From birth to 2 months postpartum | Importance of initiating and sustaining exclusive breastfeeding for low birth weight (LBW) infants, correct breastfeeding techniques (e.g., positioning, attachment, skin-to-skin contact), time and frequency of breastfeeding, nutritional needs, and weight gain monitoring for LBW infants, breast care education |
| Aysu Yıldız Karaahmet  (2022) | One in-hospital face-to-face breastfeeding training session with demonstration, followed by online counseling via interviews | One in-hospital session within 24 hours postpartum, followed by three online follow-ups (at discharge, 1 month, and 6 months postpartum) | From birth to 6 months postpartum | Importance of breastfeeding and breast milk, correct breastfeeding techniques (e.g., positioning and attachment), addressing breastfeeding problems, enhancing breastfeeding self-efficacy, and supporting exclusive breastfeeding continuation |
| Mai-Britt Hägi-Pedersen  (2022) | Video consultations with a smartphone application (PreHomeCare) for the intervention group vs. in-hospital consultations for the control group during early in-home care | Two to three planned consultations per week | From hospital admission to 1 month after discharge | Support for breastfeeding (e.g., positions, infant signals, milk expression), nutrition planning, infant weight monitoring, tube feeding guidance, and general well-being of the infant |
| Whitney Bender  (2022) | Postpartum text message-based platform with educational content and bidirectional breastfeeding support | Weekly motivational and informational text messages, plus weekly feeding inquiries, with daily responses to patient questions | From birth to 6 weeks postpartum | Educational and motivational text messages on breastfeeding, weekly inquiries about infant feeding methods, two-way text messaging for personalized support and addressing concerns, referrals to telehealth or in-person lactation support when needed |
| Daprim S Ogaji  (2021) | Mobile phone-based support with regular phone calls in addition to usual care to promote exclusive breastfeeding | Phone calls on days 7 and 14 of the first month, then monthly within 2-7 days of the infant’s monthly birthdays (average of eight calls total) | From birth to 6 months postpartum | Providing education and reminders on EBF benefits, addressing breastfeeding and infant well-being questions via phone, recording EBF termination, and introducing other foods or fluids |
| Kris Yuet-Wan Lok  (2021) | Home-based breastfeeding peer support with five home visits from trained peer supporters, supplemented by proactive WhatsApp and telephone support between visits, in addition to usual care | Five home visits at 1 week, 3 weeks, 2 months, 4 months, and 6 months postpartum, with WhatsApp/telephone contact as needed between visits | From birth to 6 months postpartum | Providing breastfeeding education, addressing breastfeeding difficulties, offering emotional and practical support through home visits, and maintaining ongoing communication via WhatsApp/telephone |
| Adam K. Lewkowitz  (2021) | Smartphone app (BreastFeeding Friend, BFF) with breastfeeding education and on-demand videos, compared with a control app with digital breastfeeding handouts, both preloaded on study-provided phones | Continuous access to the app from enrollment at ~36 weeks of gestation through 6 months postpartum | From birth to 6 months postpartum | Providing breastfeeding education, on-demand videos on breastfeeding techniques and normal infant behavior, and digital resources, with no additional phone consultations |
| Jennifer S. Cauble  (2021) | Six weekly prenatal group-based phone counseling (GBPC) sessions led by a registered dietitian (RD) and an International Board Certified Lactation Consultant (IBCLC), compared with a usual care group receiving standard pregnancy and pediatric education | Six sessions: weekly, approximately 60 minutes each | From 16–30 weeks of gestation | Introduction to breastfeeding (benefits, duration, myths), breastfeeding basics (anatomy, milk production, feeding amounts, difficulties), pumping (techniques, storage, equipment), returning to work (legal rights, challenges), introducing solids (timing, types, amounts, tips), nutrition and physical activity for breastfeeding; comprehensive manual, group discussion, weekly tasks |
| Li Ming Wen  (2020) | 3-group randomized clinical trial with nurse-led telephone support (30–60 min calls) or SMS support (2 messages/week for 4 weeks per stage) following mailed staged intervention booklets, compared to a control group receiving usual care and home safety materials | Six stages: One antenatal session, five postnatal sessions at 1, 3, 5, 7, and 10 months | From 24–34 weeks of gestation to 12 months postpartum | Promotion of healthy infant feeding practices (e.g., appropriate timing of solid food introduction, cup use), early-start tummy time, and reduction of screen time and bottle use at bedtime; based on Healthy Beginnings Trial advice |
| Drita Puharić  (2020) | 3-arm randomized controlled trial with intervention group receiving breastfeeding booklet and four proactive telephone calls, active control group receiving general pregnancy booklet and four calls, compared to standard care group with no additional materials or calls | Written breastfeeding booklet and four proactive telephone calls (oneantenatal, three postnatal at 2, 6, and 10 weeks) | From 20–32 weeks of gestation to 10 weeks postpartum | Promotion of exclusive breastfeeding via evidence-based booklet (e.g., importance of EBF, skin-to-skin contact, correct attachment) and telephone support using behavior change techniques |
| Adam K Lewkowitz  (2020) | 2-arm randomized controlled trial with intervention group receiving the BreastFeeding Friend (BFF) app (on-demand videos and education) and control group receiving an app with digital breastfeeding handouts, both provided with smartphones and internet | App usage: median 15 times (BFF) vs. 9 times (control), tracked from 36 weeks of gestation to 6 months postpartum | From 36 weeks of gestation to 6 months postpartum | BFF app: breastfeeding education (benefits, latch techniques, common challenges), newborn behavior, pumping strategies, on-demand videos, and links to support resources |
| Hiroko Hongo  (2020) | Telephone-based breastfeeding peer support by trained volunteer mothers recruited through La Leche League Japan and other channels | Proactive calls aligned with infant growth spurts; participants could initiate contact as needed; each peer supporter assisted 1-3 mothers | From hospital discharge to 4 months postpartum | Listening to concerns, emotional support, information on milk sufficiency and increasing supply, integrating breastfeeding with daily life, referrals to LLL Leaders or health professionals |
| Joanne L Clarke  (2020) | Face-to-face, telephone, and text message support by trained Infant Feeding Helpers (IFHs) using an asset-based approach | Antenatal: 1 face-to-face meeting, monthly calls/texts; Postnatal: daily calls/texts for first 2 weeks, decreasing to monthly by 5 months | 30 weeks of gestation to 5 months postpartum | Woman-centered support, social support, restructuring social environment, co-production of a Genogram, and information on local feeding resources |
| J. A. Unger  (2018) | 1-way or 2-way SMS messaging via Mobile WACh platform; messages tailored to pregnancy stage and participant profile | Weekly messages from enrollment to 12 weeks postpartum | From enrollment (mean gestational age ~26 weeks) to 12 weeks postpartum | Educational and motivational SMS messaging on exclusive breastfeeding (EBF), contraception, antenatal care (ANC), and immunizations; 2-way arm included nurse interaction and participant-initiated SMS messaging |
| Archana Patel  (2018) | Weekly cell phone counseling by certified lactation counselors + daily text messages in local language; phones and recharge vouchers provided | One counseling call/week (from third trimester to 6 months postpartum); One text message /day throughout | From the third trimester (32-36 weeks) to 6 months postpartum | Counseling on breastfeeding practices, maternal nutrition, antenatal care (ANC), infant and young child feeding (IYCF), and immunizations; real-time problem-solving; text message reinforcement; emergency counselor contact via speed dial |
| Jenny Ericson  (2018) | Daily proactive telephone calls from the NICU breastfeeding support team (BST) | Daily calls for up to 14 days; mothers could choose sparser calls or stop them | From hospital discharge to 14 days postpartum | Person-centered breastfeeding support: mothers encouraged to discuss their concerns, with a focus on their needs |
| Yanhong Gu  (2016) | Theory of Planned Behavior (TPB)-based intervention: individual instruction + group education + telephone counseling | In-hospital: 1 individual instruction (day 1) + 1 group session (day 2); Post-discharge: Two calls/week (weeks 1–2), one call/week (weeks 3–6), one call every 2 weeks (6 weeks–3 months), intensified weekly calls around 4 months then 1 call every 2 weeks (4–6 months) | From birth to 6 months postpartum | Education on breastfeeding benefits and importance, correct breastfeeding techniques, problem-solving (e.g., breast pain, engorgement), family involvement for support, preparation for work (e.g., breast milk expression, storage, workplace management) |
| Ganga L. Srinivas  (2015) | Low-intensity peer counseling by a trained breastfeeding peer counselor via telephone and in-clinic contact | Prenatal: contact between 28 weeks’ gestation and 1 week pre-delivery; Postnatal: within 3–5 days post-delivery, weekly to 1 month, every 2 weeks to 3 months, once at 4 months | From late pregnancy (~28 weeks) to 4 months postpartum | Basic breastfeeding assistance (e.g., latch, position, milk transfer assessment), encouragement to meet personal breastfeeding goals, support for breastfeeding self-efficacy |
| Norzakiah Mohd Tahir  (2013) | Telephone lactation counseling by certified lactation counselors | Twice a month postpartum (expected total: 12 calls per mother) | From birth to 6 months postpartum | Support for exclusive breastfeeding, guidance on breastfeeding techniques, addressing breastfeeding problems, and emotional support; guided by the WHO lactation management and counseling module |
| Emma Malchau Carlsen  (2013) | Telephone-based breastfeeding advisory support by a certified lactation consultant | Three calls in the first month, every 2 weeks until 8 weeks postpartum, monthly thereafter until 6 months; minimum 9 calls if breastfeeding continued | Birth to 6 months postpartum | Structured questions on physical and psychological breastfeeding aspects, well-being of mother and child, advice on difficulties, solutions, knowledge assessment and reinforcement, tracking feeding status |
| Linda C. Pugh  (2010) | Breastfeeding Support Team (BST): hospital visits, home visits, scheduled telephone support, and 24/7 pager access | Daily hospital visits until discharge; 2 home visits in the first week, 1 at 4 weeks; telephone call every 2 weeks through 24 weeks; 24/7 pager access | From delivery to 24 weeks postpartum | Education on breastfeeding, symptom management (fatigue, nipple pain), psychosocial support, infant assessment, encouragement, problem-solving, linkage to community/pediatric services, additional support as needed |
| Maya Bunik  (2010) | Daily telephone support by bilingual nurses using scripted protocols | One call/day for 14 days postpartum | From hospital discharge to 2 weeks postpartum | Education on colostrum benefits, latch, engorgement, milk supply assessment, sore nipples, thrush, breastfeeding duration, crying causes, cultural issues, support resources, maternal illness, postpartum depression, medications, pumping, milk storage, return to work/school, growth spurts, referrals for lactation/medical issues |
| Karen A. Bonuck  (2006) | Lactation consultant (LC) intervention with prenatal meetings, postpartum hospital and/or home visits, and telephone support as needed | Prenatal: Two meetings (1 in-clinic or home); Postpartum: One hospital or home visit; Telephone: as needed up to 12 months | From prenatal care to 12 months postpartum | Prenatal: breastfeeding education; Postnatal: breastfeeding support, addressing feeding issues, maternal support |
| Karen A. Bonuck  (2005) | Lactation consultant intervention, including two prenatal meetings, postpartum hospital visit and/or home visits, and telephone calls, in addition to standard care | Two prenatal meetings; postpartum hospital visit (25% received); home visits (49% received); telephone calls weekly near expected delivery date and multiple times postnatally up to 12 months | Up to 52 weeks postpartum | Prenatal and postnatal education and support: skills-based breastfeeding assistance (e.g., latch and positioning), encouragement of breastfeeding confidence, addressing barriers such as public nursing or returning to work/school |
| Forster DA (2019) | Proactive telephone-based peer support (mother-to-mother) | Initial call within 1-3 days postpartum, followed by weekly calls as needed, averaging six calls per mother (median 11 calls for those supported beyond 4 weeks) | From birth to 26 weeks (6 months) postpartum | Emotional support, encouragement, sharing breastfeeding experiences, addressing breastfeeding challenges, providing reassurance, and promoting breastfeeding continuation through peer (mother-to-mother) support. |
| Fan HSL (2022) | Online instant messaging peer support group via WhatsApp | Weekly prompts by peer counselors, with ad-hoc responses to participants' questions and discussions | From recruitment (antenatal period) to 6 months postpartum | Emotional, informational, and appraisal support; sharing breastfeeding experiences, providing advice, answering questions, and encouraging discussion on breastfeeding-related issues. |
