# Supplementary figures and images for: Effects of remote breastfeeding guidance on breastfeeding rates and neonatal health: a systematic review and meta-analysis
Source: Front Public Health. 2026 May 1;14:1696927. doi: 10.3389/fpubh.2026.1696927 (PMC13176203; doi:10.3389/fpubh.2026.1696927)

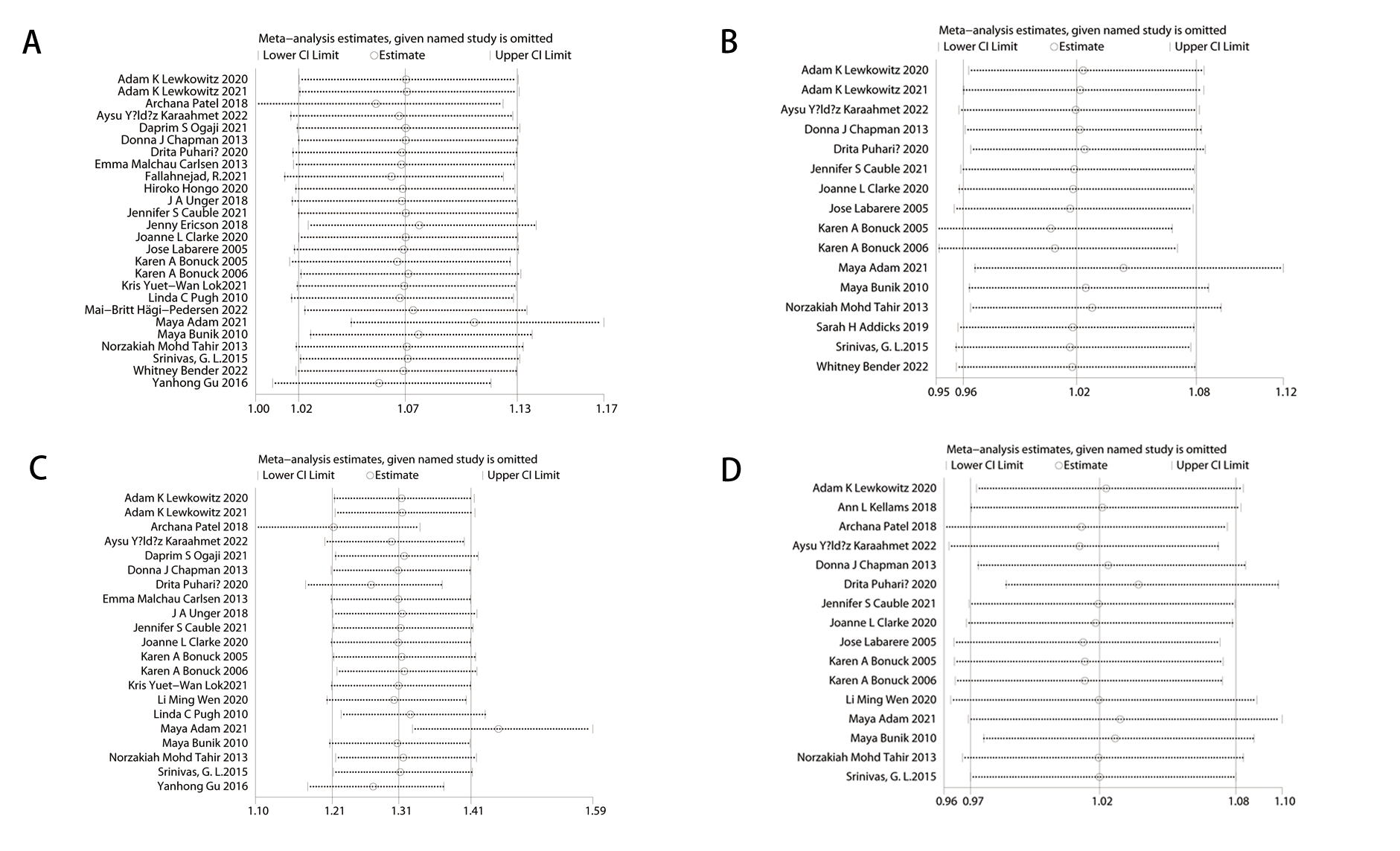

Supplement: Supplementary Figure S1 — Sensitivity analysis of the meta-analysis. (A) Exclusive breastfeeding at 3 months; (B) any breastfeeding at 3 months. (C) Exclusive breastfeeding at 6 months; (D). Any breastfeeding at 6 months. [file Image_1.TIF]

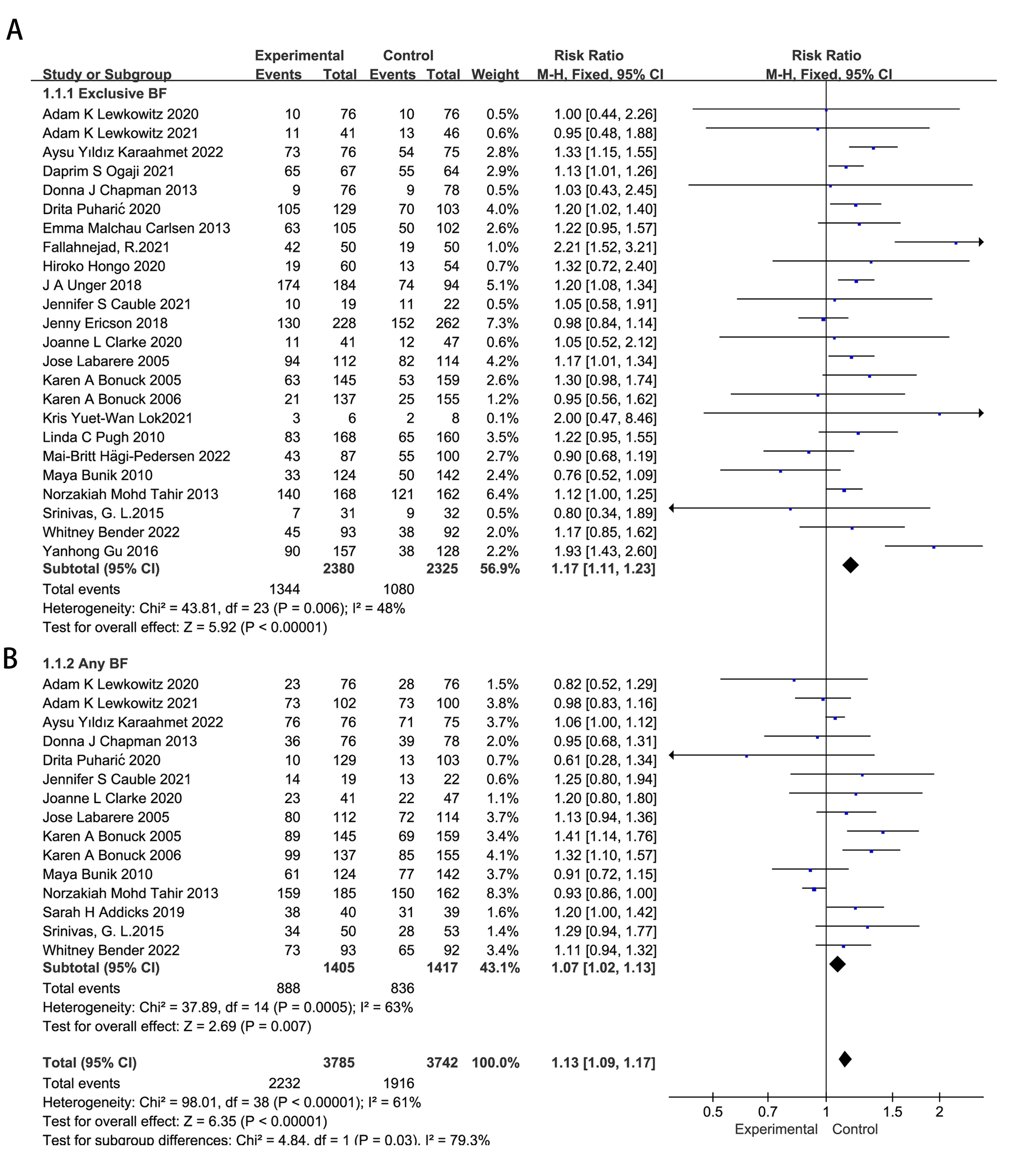

Supplement: Supplementary Figure S2 — Effects of remote breastfeeding guidance on neonatal feeding practices at 3 months. (A) Exclusive breastfeeding; (B) Any breastfeeding. RR, risk ratio; CI, confidence interval; Random, random effects model; Fixed, fixed effects model. [file Image_2.TIF]

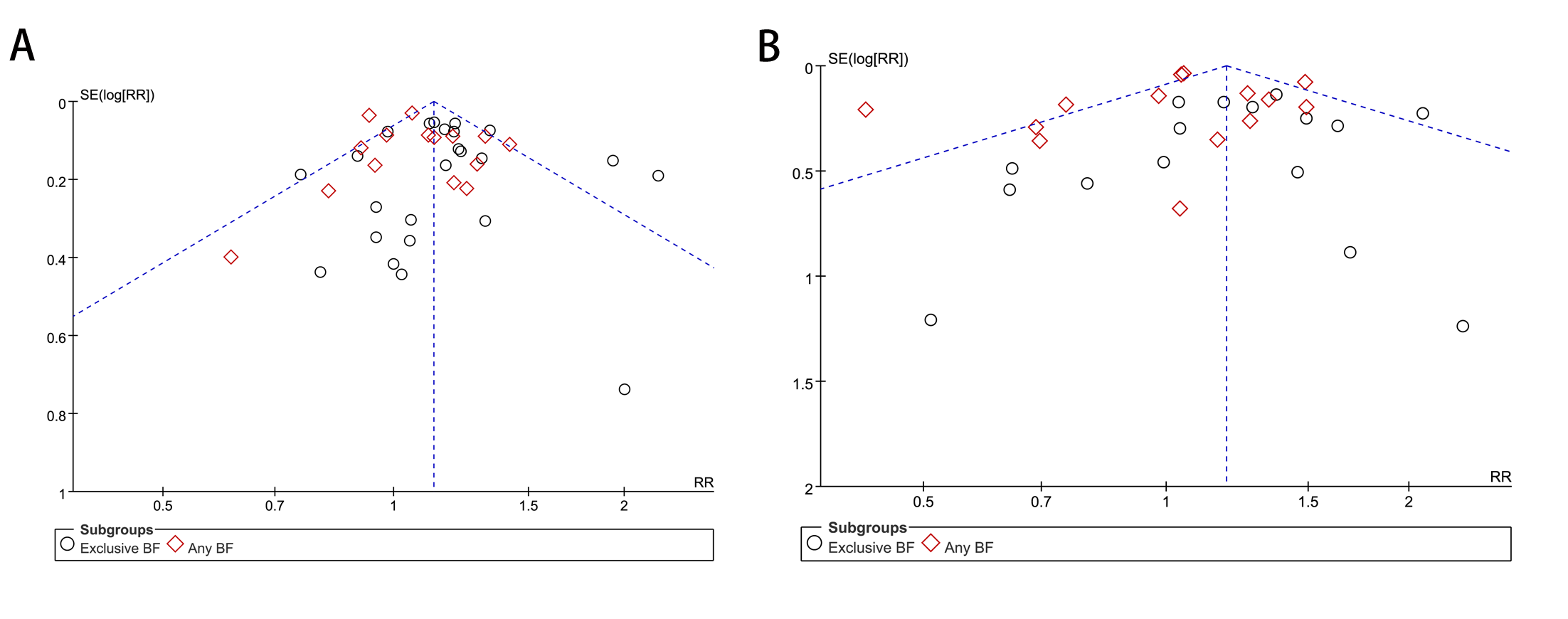

Supplement: Supplementary Figure S3 — Funnel plot of remote breastfeeding guidance at 3 months (A) and 6 months (B). [file Image_3.TIF]

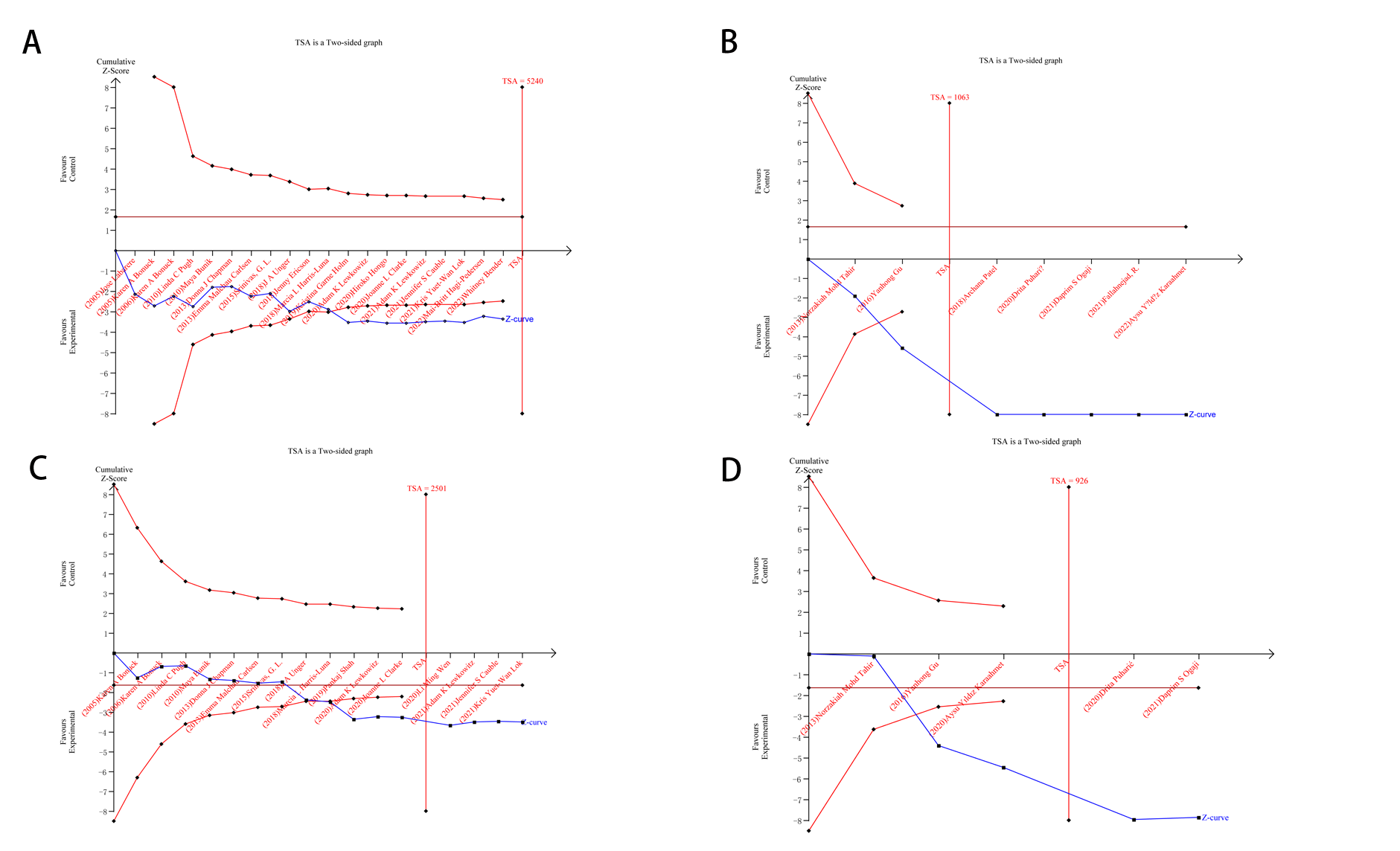

Supplement: Supplementary Figure S4 — TSA analysis of the meta-analysis. (A) Exclusive breastfeeding at 3 months; (B) any breastfeeding at 3 months. (C) Exclusive breastfeeding at 6 months; (D) Any breastfeeding at 6 months. [file Image_4.TIF]

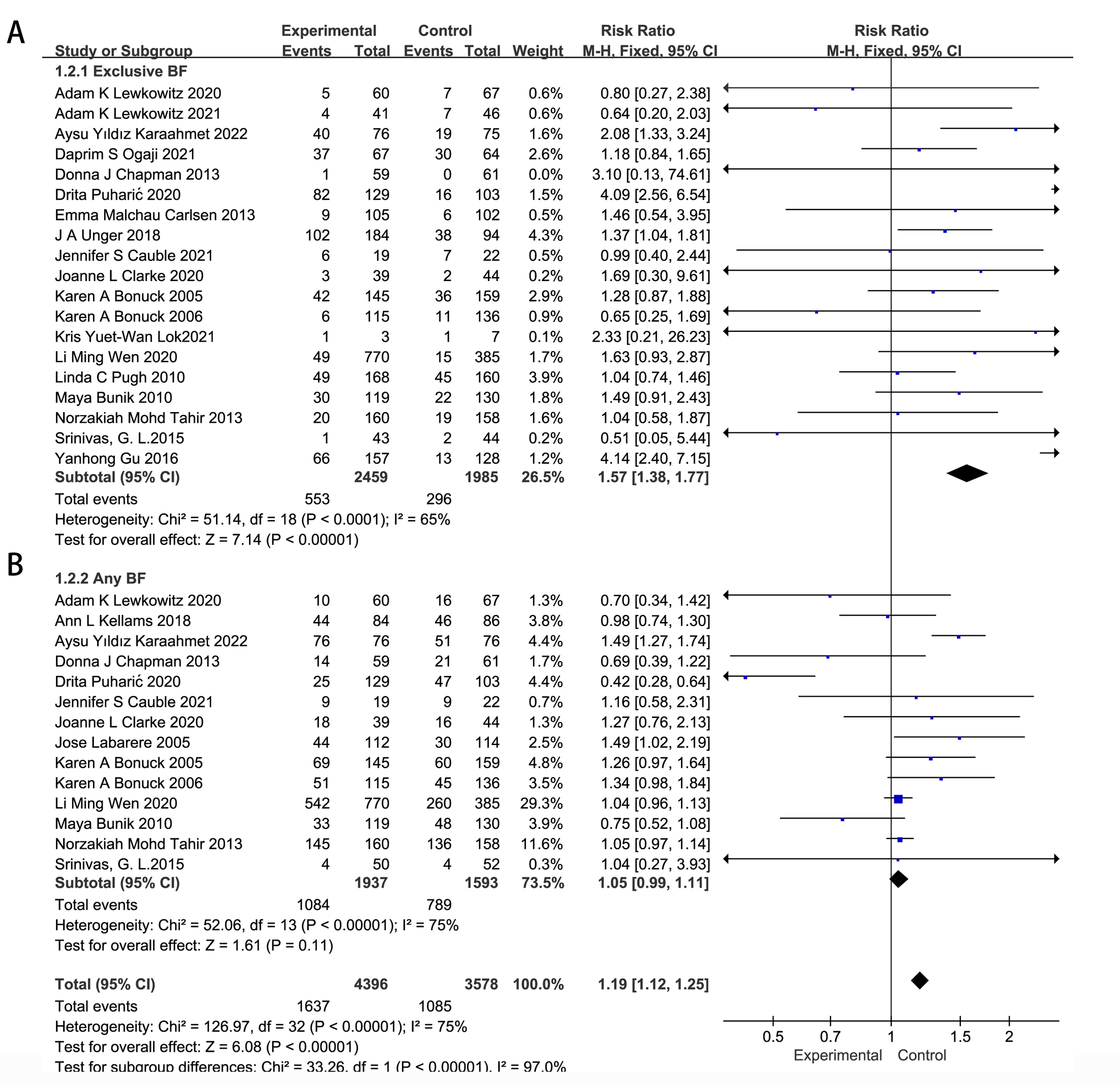

Supplement: Supplementary Figure S5 — Effects of remote breastfeeding guidance on neonatal feeding practices at 6 months. (A) Exclusive breastfeeding; (B) Any breastfeeding. RR, risk ratio; CI, confidence interval; Random, random effects model; Fixed, fixed effects model. [file Image_5.TIF]
